# Expectations and perception of cancer treatment goals in previously untreated patients. The EXPECT trial

**DOI:** 10.1007/s00520-020-05826-x

**Published:** 2020-11-07

**Authors:** Christoph Minichsdorfer, O. Zeller, M. Kirschbaum, A. S. Berghoff, R. Bartsch

**Affiliations:** Division of Oncology, Department of Medicine 1, Medical University of Vienna, Waehringer Guertel 18-20, 1090 Vienna, Austria

**Keywords:** Expectations and perceptions, Chemotherapy, Treatment goals, Informed consent, Side effects

## Abstract

**Purpose:**

Misconceptions regarding activity and toxicity of therapeutic interventions are common among cancer patients. There is little knowledge about the factors that contribute to a more realistic perception by patients.

**Methods:**

This pilot study was designed as a prospective questionnaire survey and included 101 therapy-naïve patients treated at the Division of Oncology, Medical University of Vienna. After obtaining written informed consent, patients’ expectations about treatment aims, side effects and the satisfaction with their oncologic consultation were interrogated before the first treatment cycle by questionnaires.

**Results:**

Of 101 patients, 53 (53%) were female and 67/101 (66%) were treated with curative attempt in an adjuvant or neo-adjuvant setting. The most common diagnoses were lung cancer (31%) and breast cancer (30%). Although 92% of patients were satisfied with the information given by their oncologist, palliative patients were more likely to declare that not everything was explained in an intelligible manner (*p* = 0.01). Patients with a first language other than German stated more often that their physician did not listen carefully enough (*p* = 0.02). Of 30 patients, 26 (87%) receiving chemotherapy with palliative intent believed that their disease was curable. Concerning adverse events, female patients anticipated more frequently hair loss (*p* = 0.003) and changes in taste (*p* = 0.001) compared to men. Patients under curative treatment were more likely to expect weight loss (*p* = 0.02) and lack of appetite (*p* = 0.01) compared to patients with palliative treatment intent.

**Conclusion:**

In conclusion, cancer patients were satisfied with the patient-doctor communication. This prospective study aggregated patients’ concerns on side effects and the perception of therapeutic goals in therapy-naïve patients. Of note, the majority of patients treated in the palliative setting expected their treatment to cure the disease.

**Supplementary Information:**

The online version contains supplementary material available at 10.1007/s00520-020-05826-x.

## Introduction

Patients with advanced malignancies often overestimate the benefit of chemotherapy and have wrong perceptions regarding the therapeutic intent of treatment [1–5]. Typically, discrepancies between the patients’ perception and the view of their respective oncologists can be observed. There is evidence that a significant number of palliative patients believe that their therapy has a curative intent and lack awareness about their life expectancy [5–14]. Interestingly, prognostic awareness seems to be related to a worse QoL [15]. However, selective coping strategies can alleviate a part of the problem [16].

Female patients and patients with high school or university degree are more likely to understand their diagnosis. Patients’ awareness of the treatment intent with lower education seems to be very low [1]. In addition, lower income and a lack of social support lead to wrong perceptions of treatment goals in elderly patients [17]. Of note, misconceptions about the aim of treatment are not limited to palliative patients as was shown in a study including patients with early-stage solid malignancies [7, 18].

These data prompted the question whether patients are able to participate in a joint decision-making process as actually recommended [19, 20]. In the last decades, innovative therapeutic protocols and supportive therapy have markedly improved the patient’s benefit. Additionally, awareness and acceptance of malignant diseases have improved with information about cancer readily available through the internet. However, unfiltered information can be biased and even misleading.

Given that misconception of treatment goals is common in patients with incurable malignant diseases and that limited data are available for patients in a curative setting, this prospective study was conducted to evaluate information and expectations of patients starting their first cancer treatment.

## Methods

### Patient population

Patients who started therapy at the Division of Oncology at the Medical University of Vienna were included in this prospective pilot study. After the first consultation with their medical oncologist and before the first round of therapy, patients were recruited in consecutive order. We included patients with solid cancers before the first cycle of neo-adjuvant, adjuvant or first-line palliative chemotherapy with a life expectancy > 3 months.

After obtaining written informed consent, patients were interviewed prior to therapy start. Sufficient German language skills were required. Three proficiency levels were defined by Adult Education Survey: a fair level was defined as “I can understand and use the most common everyday expressions. I use the language in relation to familiar things and situations”; a good level was defined as “I can understand the essentials of clear language and produce simple text. I can describe experiences and events”; and a proficient level corresponds to “I can understand a wide range of demanding texts and use the language flexibly” [21]. For study inclusion, the classification “fair level” was sufficient. Prior chemo- or radiotherapy was an exclusion criterion.

### Questionnaire

In the out-patient ward, patients were asked to answer a simple questionnaire with assistance from a nurse, a psychologist or a medical student when necessary. The physicians doing the survey were not involved into decision-making regarding the anticancer treatment. The patients were informed about data protection and anonymisation, which prevented to link questionnaires to specific patients.

Information obtained in the survey included the following: satisfaction with oncologist communication, sources of information used by the patient, relationship status, highest education, if their first language was German, if Austria was their country of birth, perception of treatment goals, anticipation how strong therapy may interfere with the activities of daily life and the expected frequency of therapy-related adverse events. In detail, we asked the patients whether they expect the lack of appetite, nausea/vomiting, weight loss, weariness/weakness, dizziness, pain, numbness in arms/legs, breathlessness/dyspnoea, infections, change of taste, infections of the oral mucosa, changes in emotions/sexuality, hair loss, diarrhoea and constipation. After three cycles of therapy, patients were asked which side effects occurred how frequently.

In total, there were 37 questions the patients had to answer in the first questionnaire (ESM). Questions regarding the patients’ satisfaction regarding the communication with their doctor had to be answered in a range of 1 to 4 (1 = never, 2 = seldom, 3 = sometimes, 4 = always). Questions about the patients’ expectations of treatment side effects and about treatment aims also had to be answered in a range of 1 to 4 (1 = not at all, 2 = slightly, 3 = moderately, 4 = strongly). In the context of treatment-related side effects, patients were asked to exclusively report if they expect a specific side effect to occur rather than the anticipated grade of severity. All other questions were to be answered yes or no. In addition to this questionnaire, every patient was handed out the EORTC QLQ-c30 questionnaire, a validated questionnaire focused on the quality of life in patients with cancer.

Before the third cycle of the therapy, patients were asked to answer a follow-up questionnaire to assess the side effects that had actually occurred. However, due to the low number of returned follow-up questionnaires (only 16 patients returned the follow-up questionnaire), this study part was not calculated by statistics.

Questionnaires were evaluated and peer-reviewed by staff members of the specialized psycho-oncological team of the Division of Oncology and are provided in the supplement as questionnaire part I and part II. As this is the pilot study, the questionnaire has not been validated yet. The study was approved by the local ethics committee (ECS 2153/2013).

### Statistics

Statistical analysis was done using SPSS Statistics, version 22. To describe demographic data and answers to the questionnaire, descriptive statistics were applied. To test differences between groups, the chi-square test was used. In detail, we correlated the answers to the questions about patients’ satisfaction with the conversation with their respective physicians and about their knowledge regarding treatment aims and their expectations on side effects of the planned therapy with the following factors: gender, treatment intent (curative vs. palliative), first language (German or other), participation in an oncologic support group and level of education (see below).

To compare patient’s expectations regarding the side effects of chemotherapy with the side effects actually experienced, the *t* test for paired samples was used. Due to the exploratory design and the therefore limited patient number, only bivariate analyses were conducted.

A level of statistical significance of *p* < 0.05 was considered statistically significant.

## Results

### Patients

A total number of 101 patients was included and participated in the survey. Fifty-three percent were female and 47% were male with a median age of 56.2 years. The majority of patients received chemotherapy with curative intent (66%). In total, 12% received single-agent therapy, while 68% received a combination of different cytotoxics. The remaining subjects received different antibody-chemotherapy combinations. No patient received immune-checkpoint inhibitor therapy.

German was the mother tongue of 77% of patients and 30% had at least a high school degree. Seventy percent of patients lived in a relationship, whereas 26% lived alone; 4% refused to answer this question. When asked about sources of information besides the consultation with their medical oncologists, 58% of patients stated that they were informed by relatives and friends, 21% searched for information in books and magazines; the internet was consulted by 47% patients. In addition, 34% of patients received information from the nursing stuff.

Regarding the site of primary cancer, 31% of patients were diagnosed with lung cancer and 30% with breast cancer. Other cancer subtypes included were pancreaticobiliary cancer (9%), testicular cancer (7%), colorectal cancer (4%) and malignant lymphoma (4%). The demographic data are listed in Table 1.

**Table 1:** Demographic characteristics of the included patient population

	*n* (%)
Patients	101 (100)
Sex	
Female	53 (53)
Male	48 (47)
Median age	56.2 years
Treatment intent	
Curative	67 (66)
Palliative	34 (34)
Therapy	
Mono CHT	12 (12)
Combined CHT	69 (68)
Antibody-CHT	20 (20)
Education	
High school	31 (30)
University	6 (6)
Compulsory school	64 (64)
Mother tongue	
German	78 (77)
Relationship status	
Married	71 (70)
Single	26 (26)
Not answered	4 (4)
Source of information	
Friends/relatives	59 (58)
Books/magazines	21 (21)
Internet/TV	48 (47)
Nursing staff	35 (34)
Diagnosis	
Lung cancer	31 (30)
Breast cancer	30 (29)
Pancreatic cancer	9 (9)
Testicular cancer	7 (7)
Colorectal cancer	4 (4)
Lymphoma	4 (4)
Head and neck cancer	3 (3)
Sarcoma	3 (3)
Others	10 (10)

### Patients’ assessment of doctor’s communication

In total, 94 of the 101 patients returned an answer to this question. Overall, patients rated the conversation with their respective physicians as being exceptionally good. The majority of patients (94/97, 92.2%) felt that the treating oncologist was listening to their questions and concerns (median 3.8 (95% CI 3.7–3.9). In addition, most patients (95/97, 93.1%) felt that all topics regarding their disease and therapy were explained in an intelligible manner (median 3.8; 95% CI 3.7–3.9). The majority of participants (91/95, 89.2%) felt that they had received all necessary information (median 3.8; 95% CI 3.7–3.9). Likewise, most patients (85/95, 83.3%) felt that they were encouraged by their physician to ask questions regarding their treatment (median 3.5; 95% CI 3.3–3.7).

Patients’ educational level and gender were not correlated with the communication quality. However, when patients were analysed according to their treatment intent (palliative vs. curative), palliative patients were less convinced that comprehensible information was provided (*p* = 0.01) (Table 2). In addition, patients with a first language other than German felt significantly more often that their doctor did not listen to them adequately (*p* = 0.02).

**Table 2:** Perception and satisfaction of patients with their doctor’s communication. Palliative and curative patients were compared with a chi^2^ and Fisher’s exact test; *p* < 0.05 was assumed to be significant. *CI*, confidence interval; *SD*, standard deviation

					*n*	Mean	95% CI	SD	*p*
Did your doctor listen carefully to your questions?				Total	94	3.8	3.7–3.9	0.5	
				Curative	65	3.8	3.7–3.9	0.5	
				Palliative	29	3.7	3.5–3.9	0.5	0.5
Did your doctor explain everything intelligible?				Total	94	3.8	3.7–3.9	0.5	
				Curative	65	3.9	3.8–3.9	0.3	
				Palliative	29	3.6	3.3–3.8	0.6	*0.01*
Did your doctor give you sufficient information?				Total	94	3.8	3.7–3.9	0.5	
				Curative	65	3.9	3.8–4	0.4	
				Palliative	29	3.7	3.4–3.9	0.7	0.1
Did your doctor encourage you to ask questions?				Total	94	3.5	3.3–3.7	0.8	
				Curative	65	3.5	3.3–3.7	0.7	
				Palliative	29	3.5	3.2–3.8	0.8	0.7

### Patients’ perception of treatment aims

**Fig. 1:**
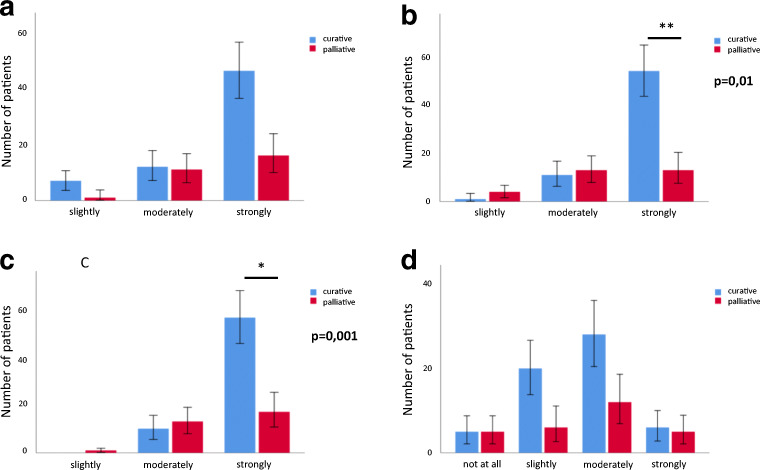
The perception of treatment goals discerned between palliative and curative patients. **a** Will the chemotherapy alleviate cancer-related symptoms? **b** Will the therapy cure your malignant disease? **c** Will the therapy help you to live longer? **d** Will the therapy affect your daily activities? Chi^2^ tests were calculated to show significant differences in the perception of treatment goals between palliative and curative patients. A *p* value < 0.05 was assumed to be statistical significant

Patients in general have an optimistic belief regarding the therapeutic goals of their treatment (Table 3). Palliative patients, however, had a significantly lower expectation to be cured (median: 3.8 vs. 3.5; *p* = 0.004) or to live longer as a result of the administered chemotherapy (median: 3.8 vs. 3.2; *p* = 0.001). Still, the majority of palliative patients (26/30, 87%) moderately or strongly believed that the therapy would cure their disease (Fig. 1). Most patients expected that the therapy will help to alleviate cancer-related symptoms (85/93, 91.4%). Consequently, impairments in daily and social activities (hobbies, shopping, cooking or contacts) were only moderately anticipated (51/87, 58.6%) (Table 3 and Fig. 1).

**Table 3:** Perception of treatment goals of chemotherapy-naive patients. Palliative and curative patients were compared with a chi^2^ test, *p* < 0.05 was assumed to be significant. *CI*, confidence interval; *SD*, standard deviation

					*n*	Mean	95% CI	SD	*p*
Will the therapy help you to live longer?				Curative	58	3.8	3.7–3.9	0.4	
				Palliative	25	3.5	3.2–3.8	0.6	*0.004*
Will the therapy cure your malignant disease?				Curative	58	3.8	3.7–3.9	0.4	
				Palliative	25	3.2	2.9–3.5	0.7	*0.0001*
Will the therapy alleviate cancer related symptoms?				Curative	58	3.6	3.4–3.8	0.7	
				Palliative	25	3.5	3.3–3.8	0.6	0.08
Will the therapy affect your daily activities?				Curative	58	2.6	2.4–2.8	0.8	
				Palliative	25	2.7	2.3–3.0	0.9	0.3

### Patients’ expectations of the occurrence of adverse events

Females were more likely to expect hair loss (*p* = 0.003) and changes in taste (*p* = 0.001). Of note, hair loss was the most frequently anticipated side effect in women, followed by fatigue, changes of taste, nausea and appetite loss (median 3.3 for hair loss, 2.9 for fatigue, 2.5 for changes of taste, 2.4 for nausea and appetite loss respectively) (Table 4). For male patients, fatigue, hair loss, appetite loss, nausea, weight loss and changes in emotions/sexuality were most troublesome (median 2.7 for fatigue, 2.5 for hair loss, 2.1 for loss of appetite, nausea, weight loss and 2.0 for changes in emotions/sexuality) (Table 4).

**Table 4:** Patient expectations regarding the occurrence of possible side effects. Female and male patients were compared and a chi^2^ and Fisher’s exact test were performed; *p* < 0.05 was assumed statistical significant. *CI*, confidence interval; *SD*, standard deviation

Adverse event		Mean	95% CI	SD	*p*			Mean	95% CI	SD	*p*
Appetite loss	Female	2.4	2.2–2.7	0.8		Dizziness	Female	2	1.8–2.3	0.8	
	Male	2.1	1.8–2.4	0.9	0.09		Male	1.9	1.6–2.2	0.8	0.12
Change of taste	Female	2.5	2.2–2.8	0.9		Pain	Female	1.9	1.7–2.1	0.8	
	Male	1.8	1.6–2.0	0.7	*0.001*		Male	1.6	1.4–1.9	0.7	0.08
Nausea/vomiting	Female	2.4	2.1–2.7	0.9		Neuropathy	Female	1.7	1.5–2	0.7	
	Male	2.1	1.8–2.4	0.8	0.07		Male	1.7	1.4–2.1	0.9	0.07
Weight loss	Female	2.2	2.0–2.4	0.7		Dyspnoea	Female	1.5	1.3–1.7	0.7	
	Male	2.1	1.8–2.4	0.8	0.35		Male	1.6	1.3–1.9	0.8	0.53
Fatigue	Female	2.9	2.6–3.2	0.9		Changes in emotions/sexuality	Female	2.3	2.0–2.7	1.1	
	Male	2.7	2.4–3.0	0.9	0.49		Male	2	1.7–2.3	0.9	0.19
Hair loss	Female	3.3	3.0–3.7	1		Diarrhoea	Female	2	1.8–2.2	0.7	
	Male	2.5	2.1–2.9	1.2	*0.003*		Male	1.8	1.5–2.1	0.8	0.48
Infection/fever	Female	1.7	1.5–2.0	0.8		Constipation	Female	1.9	1.6–2.2	0.9	
	Male	1.5	1.2–1.7	0.7	0.08		Male	1.7	1.5–2.0	0.7	0.34
						Mucositis	Female	2.1	1.8–2.3	0.9	
							Male	1.7	1.4–2.0	0.8	0.12

Compared with palliative patients, curative patients anticipated more often a lack of appetite (*p* = 0.02), weight loss (*p* = 0.01) and mucositis (*p* = 0.002). Age had a minimal influence on expectation of side effects, but younger patients (< 65 years) expected mucositis more often than elderly patients (≥ 65 years) (*p* = 0.038). Educational status and first language other than German had no influence on the expectation of treatment-related adverse events.

## Discussion

It is known from previous studies that patients with cancer often have wrong perceptions of their treatment goals and are likely to underestimate the occurrence of side effects. The majority of these studies were performed in patients with incurable disease. As a consequence, we aimed to include a majority of patients under curative treatment as this collective is underrepresented in the current literature. This study aimed to evaluate the patients’ level of information regarding their disease and their treatment and to assess the patient satisfaction regarding communication with their oncologists.

Patients were asked to rate the quality of the conversation with the doctor prior to treatment initiation and most patients stated that their oncologist listened to their questions, explained the situation intelligibly, provided sufficient information and encouraged patients to ask questions (Table 2). Therefore, the doctors’ attention and conversational skills met the patients’ expectations, in line with findings of other surveys concerning this topic [7, 9]. While this suggests a high level of satisfaction, we cannot estimate the baseline level of expectation patients might have regarding the conversation with their respective oncologist. Of note, the level of education had no influence on the results.

Other studies suggested a significant difference regarding knowledge about the diagnosis and the treatment intent when different educational levels and social status were compared [5, 6, 11, 17]. In contrast, no significant difference was observed in this study. The majority of other analysis, however, divided patients into more than two groups (e.g. elementary school, high school and college-educated) [6]. Similar surveys, where educational status was divided into two groups, found no differences as well [9].

There were two relevant factors associated with the patient’s opinion on the quality of the communication with their physicians. Patients whose first language was not German felt that their doctor did not listen to them with due attention, which was not experienced by subjects born in Austria. Second language learners may have significant deficits in reading and language comprehension, which is further aggravated in individuals with a low socioeconomic background and people using primarily their first language at home [22]. However, we were not able to assess the socioeconomic background nor which language was primarily spoken at home. Furthermore, it is well described that culturally and linguistic diverse (CALD) patients are often excluded from clinical trials [23, 24]. A retrospective study in 19,543 cancer patients found that trial inclusion was significantly lower in CALD patients (OR: 0.80, 95% CI 0.69–0.91; *p* = 0.001). Another survey among 301 medical oncologists and surgeons in California reported that 57% perform less patient-centred treatment discussion with patients with limited English proficiency. The use of professional interpreters was associated with a significantly higher rate of patient-centred treatment decision (OR: 0.47, 95% CI: 0.26–0.85). The patient collective included in our study had at least a level of second language skills providing enough understanding for familiar tasks and situations or basic everyday language. In the context of complex medical decision-making, however, this patient population may be in need of more careful and mindful explanations.

Most of the other medical studies in this field of research assessed their patients’ ethnic group and/or place of birth; in a large cohort of 1193 palliative patients with CRC or lung cancer, Weeks and co-workers showed that inaccurate beliefs towards treatment goals were higher in non-white and non-Hispanic patients (OR 2.9, 95% CI 1.8–4.8 and 2.8, 95% CI 1.3–2.4) [5]. A similar result about patient-oncologist discordance about survival prognosis rating was found by Gramling and co-workers with 68% discordance in a total of 236 patients and 95% discordance in the non-white cohort with advanced disease [14]. In the light of our results, we would suggest that special care is indicated when discussing treatment strategies with patients with a migration background.

Patients receiving chemotherapy with palliative intent reported significantly less often that their oncologists explained everything intelligibly to them (Table 2). This raises the questions if palliative and curative patients have differing demands as it appears that patients with an incurable disease have a higher need of information regarding their therapy. Indeed, bad patient-doctor communication is frequently reported as a major factor for poor care in palliative patients [25–27]. Especially when it comes to providing information about dismal prognoses or reassuring that the information given was fully understood, deficits are obvious [26]. Indeed, a caregiver’s optimistic view towards a therapy is often misinterpreted and can lead to misunderstanding of prognosis and treatment goals [28]. In a nicely conducted study by Chou and colleagues in 26 patients with advanced cancer, the communication behaviour of oncologists was analysed in detail. Especially in difficult situations when the extent of the malignant disease or the prognosis was addressed, oncologists tended to use ambiguous language and vague expressions, which can easily be misunderstood by patients [29].

As to patients’ knowledge about the treatment aims, our results were comparable to other studies. While patients receiving treatment with curative intent were significantly more likely to strongly believe that chemotherapy would help them to live longer and cure their disease, 87% of patients receiving treatment with palliative intent thought the same.

Similar observations have been made by other groups with 69–81% of palliative patients believing in the curative intention of their treatment [5, 10, 12]. While more recent studies reported lower rate of misinterpretation of treatment goals [6, 7, 11], a small study of 30 palliative patients presented at the ASCO 2015 by Faricy-Anderson and co-workers showed that approximately 50% of patients had a wrong perception of treatment goals. This continuing misinterpretation of treatment aims may be even more troublesome today, when patients should be involved in shared decision-making process, because our data indicate the need for significant improvements. For example, a take-home booklet serving as decision-making aid led to a significantly better understanding of treatment goals, prognosis and the risk/benefit ratio of treatment in patients with colorectal cancer [11]. This misconception of treatment goals by our patients may be explained by coping strategies such as denial, which are well described in this patient collective [16, 30]. On the other hand, poor communication skills by medical oncologists cannot be excluded as a possible factor for this misinterpretation.

Functional impairments influencing the activities of daily life were only moderately anticipated in our cohort. This is a little surprising as functional impairments are frequently described in cancer patients, especially in the context of cancer-related fatigue [31, 32]. There are limited data available for the expectations of functional impairments in relation to chemotherapy. Either our patients did not believe that their therapy might influence their activities of daily life, or they were not thoroughly informed about possible impairments, again highlighting a possible lack of communication.

Regarding side effects, patients most often expected hair loss, nausea, fatigue and appetite loss. This is in line with the findings of Lorusso and colleagues reported in an Italian patient cohort [33]; in our study, women expected hair loss more often than men (Table 4). These authors also reported statistically significant differences between gender in the expectation of nausea and diarrhoea [33], while in our cohort, there was no gender-specific difference regarding these side effects. Nevertheless, significantly more patients under curative treatment expected lack of appetite, weight loss and mucositis, an observation potentially caused by a higher symptom burden in patients with advanced disease resulting in a more positive attitude towards chemotherapy. Interestingly, our data showed that there were only minor differences related to age and no statistically significant differences between patients according to educational status or first language.

### Limitations

It has to be taken into account that due to the limited number of patients and the design as a pilot study with no validation of the questionnaire, no multivariate analyses were conducted and the results of the bivariate analysis have to be interpreted with caution. Moreover, we have to state that only 26 patients were treated with a palliative treatment intent. Consequently, this low number might have biased these results and led to this high number of misinterpretation of treatment goals. Furthermore, after 3 months, we had planned to assess the side effects with the same questionnaire in order to compare the experienced side effects with those expected at the beginning of therapy. At inclusion to the study, the follow-up questionnaires were handed out to the patients and asked to return the filled in questionnaires after 3 cycles of therapy. Unfortunately, only 16 questionnaires were returned. Therefore, we decided to omit these data. The introduction of checkpoint inhibitors (CI) has changed the therapeutic spectrum for various malignancies and changed the spectrum of side effects. However, no patients with CI therapy were included.

## Conclusion

This study prospectively analysed patient’s expectations of side effects and perceptions about treatment goals. In summary, our results show that patients with a first language other than German more often felt that their doctor did not listen to them carefully and patients with incurable disease were more likely to state that not everything was explained to them intelligible. Overall, the communication between oncologist and patient was judged highly favourably by cancer patients starting chemotherapy. Women were more likely to expect hair loss and change of taste compared to men and curative patients expected weight loss and lack of appetite to a significantly higher extent than patients with advanced disease. Most importantly, > 80% of patients receiving chemotherapy with palliative intent believed that the upcoming treatment offered the chance of curing their disease.

Even though patients were mostly satisfied with the doctor-patient communication, wrong perceptions of treatment goals are common in patients with advanced disease. This indicates an urgent need for improved communication to allow for joint decision-making especially in patients with advanced stages of their disease and in CALD patients.

To be considered in clinical practice, the perception of side effects may differ markedly between sexes and between patients in palliative and curative treatment settings.

## Electronic supplementary material

ESM 1 (PDF 84.3 kb)

ESM 2 (PDF 53.5 kb)
