# Supplementary material for: Expectations and perception of cancer treatment goals in previously untreated patients. The EXPECT trial
Source: Support Care Cancer. 2020 Nov 7;29(7):3585–92. doi: 10.1007/s00520-020-05826-x (PMC8163685; doi:10.1007/s00520-020-05826-x)
Supplement: Supplementary file 1 — (PDF 84.3 kb) [file 520_2020_5826_MOESM1_ESM.pdf]

EXPECT Fragebogen (Teil 1):

Geburtsjahr:_____ heutiges Datum:_____
Größe: ____ Gewicht: _____

Wie oft hat ihr Arzt,

Ihnen sorgfältig zugehört

nie (1), *selten* (2), *manchmal* (3), *immer* (4)	1	2	3	4

Ihnen Dinge für Sie verständlich erklärt

nie (1), *selten* (2), *manchmal* (3), *immer* (4	1	2	3	4

Ihnen die Information über Ihre Erkrankung und Therapie gegeben die Sie wollten

nie (1), *selten* (2), *manchmal* (3), *immer* (4	1	2	3	4

Sie ermutigt krankheitsbezogene Fragen zu stellen?

nie (1), *selten* (2), *manchmal* (3), *immer* (4	1	2	3	4

Nachdem Sie mit Ihrem Arzt über Ihrer Erkrankung und Therapie gesprochen haben, wie sehr glauben Sie, wird Ihnen die Chemotherapie

helfen länger zu leben?

gar nicht (1), *etwas* (2), *mäßig* (3), *stark* (4)	1	2	3	4

helfen Ihre Krebserkrankung zu heilen?

gar nicht (1), *etwas* (2), *mäßig* (3), *stark* (4)	1	2	3	4

helfen Probleme durch Ihre Krebserkrankung zu bekämpfen?

gar nicht (1), *etwas* (2), *mäßig* (3), *stark* (4)	1	2	3	4

Wie sehr glauben Sie, dass Sie wird die Chemotherapie Ihre gewohnten Alltagstätigkeiten (Einkaufen, Hobbies, soziale Kontakte) beeinträchtigen?

gar nicht (1), *etwas* (2), *mäßig* (3), *stark* (4)	1	2	3	4

Herr christoph minichsdorfer

Welche Nebenwirkungen erwarten Sie sich durch die geplante Chemotherapie?

gar nicht (1), *etwas* (2), *mäßig* (3), *stark* (4)

Appetitlosigkeit…	1	2	3	4
Übelkeit/Erbrechen…	1	2	3	4
Gewichtsverlust…	1	2	3	4
Müdigkeit/Schwäche…	1	2	3	4
Schwindel…	1	2	3	4
Schmerzen…	1	2	3	4
Gefühlsstörungen an Armen und/oder Beinen…	1	2	3	4
Luftnot/Dyspnoe…	1	2	3	4
Infektionen…	1	2	3	4

	ja	nein
Sind Sie verheiratet oder in einer Lebensgemeinschaft?		
Leben Sie alleine?		
Ist Deutsch Ihre Muttersprache?		
Wurden Sie in Österreich geboren?		
Nehmen Sie an einer onkologischen Selbsthilfegruppe teil?		

Welche Ausbildung haben Sie abgeschlossen?	ja	nein
Pflichtschule		
Lehre		
Matura		
Universität/Fachhochschule		

Verbringen Sie mehr als 50% des Tages liegend?		

Herr christoph minichsdorfer
