# Supplementary material for: Expectations and perception of cancer treatment goals in previously untreated patients. The EXPECT trial
Source: Support Care Cancer. 2020 Nov 7;29(7):3585–92. doi: 10.1007/s00520-020-05826-x (PMC8163685; doi:10.1007/s00520-020-05826-x)
Supplement: Supplementary file 2 — (PDF 53.5 kb) [file 520_2020_5826_MOESM2_ESM.pdf]

EXPECT Fragebogen (Teil 2):

Geburtsjahr:_____ heutiges Datum:_____

Größe: ____ Gewicht: _____

1) Welche Nebenwirkungen hatten Sie während der Chemotherapie?

gar nicht (1), *etwas* (2), *mäßig* (3), *stark* (4)

Appetitlosigkeit…	1	2	3	4
Übelkeit/Erbrechen…	1	2	3	4
Gewichtsverlust…	1	2	3	4
Müdigkeit/Schwäche…	1	2	3	4
Schwindel…	1	2	3	4
Schmerzen…	1	2	3	4
Gefühlsstörungen an Armen und/oder Beinen…	1	2	3	4
Luftnot/Dyspnoe…	1	2	3	4
Infektionen…	1	2	3	4
Geschmacksveränderungen	1	2	3	4
Entzündung der Mundschleimhaut	1	2	3	4

2)) Konnten Sie Ihren gewohnten Alltag ohne Probleme fortsetzten?

	ja	nein	Ich weiß nicht
Arbeiten im Haushalt…			
Einkaufen…			
Arztbesuche…			
Hobbies…			
Mit Freunden treffen…			

3) Hat sich Ihr Gesundheitszustand verbessert?

gar nicht (1), *etwas* (2), *mäßig* (3), *stark* (4)	1	2	3	4

4) Hat sich Ihr Gesundheitszustand verschlechtert?

gar nicht (1), *etwas* (2), *mäßig* (3), *stark* (4)	1	2	3	4

Herr christoph minichsdorfer
